# A XANES study of Cu adatom coordination on Fe_3_O_4_(001)

**DOI:** 10.1039/d5cc04046a

**Published:** 2026-09-03

**Authors:** Fulden Eratam, Lena Haager, Benedikt P. Klein, Matthew A. Stoodley, Matthias Meier, Alexei Preobrajenski, Stephan Appelfeller, Gareth S. Parkinson, David A. Duncan

**Affiliations:** a Diamond Light Source, Harwell Science and Innovation Campus Didcot OX11 0DE UK david.duncan@nottingham.ac.uk; b Institute of Applied Physics, Vienna University of Technology Wiedner Hauptstrasse 8-10/134 1040 Vienna Austria; c Department of Chemistry, University of Warwick Gibbet Hill Road Coventry CV4 7AL UK; d Faculty of Physics, University of Vienna Kolingasse 14-16 1090 Vienna Austria; e MAX IV Laboratory, University of Lund Fotongatan 2 224 84 Lund Sweden; f School of Chemistry, University of Nottingham, University Park Nottingham NG7 2RD UK

## Abstract

We report XANES L-edge measurements of a precisely defined Cu_1_/Fe_3_O_4_(001) model single-atom catalyst. The spectrum acquired from the 2-fold coordinated Cu adatoms strongly resembles that of bulk Cu_2_O, with the addition of a pre-edge feature at 931 eV. We show that this feature arises from the adsorption geometry of the Cu adatom and is enhanced by the structural deformation induced by CO adsorption.

## Abstract 2

The local coordination environment is known to influence the catalytic properties of so-called “single atom” catalysts (SACs). We report XANES L-edge measurements of Cu adatoms on a well-defined Fe_3_O_4_(001) template, before and after CO ligation.

## Graphical abstract

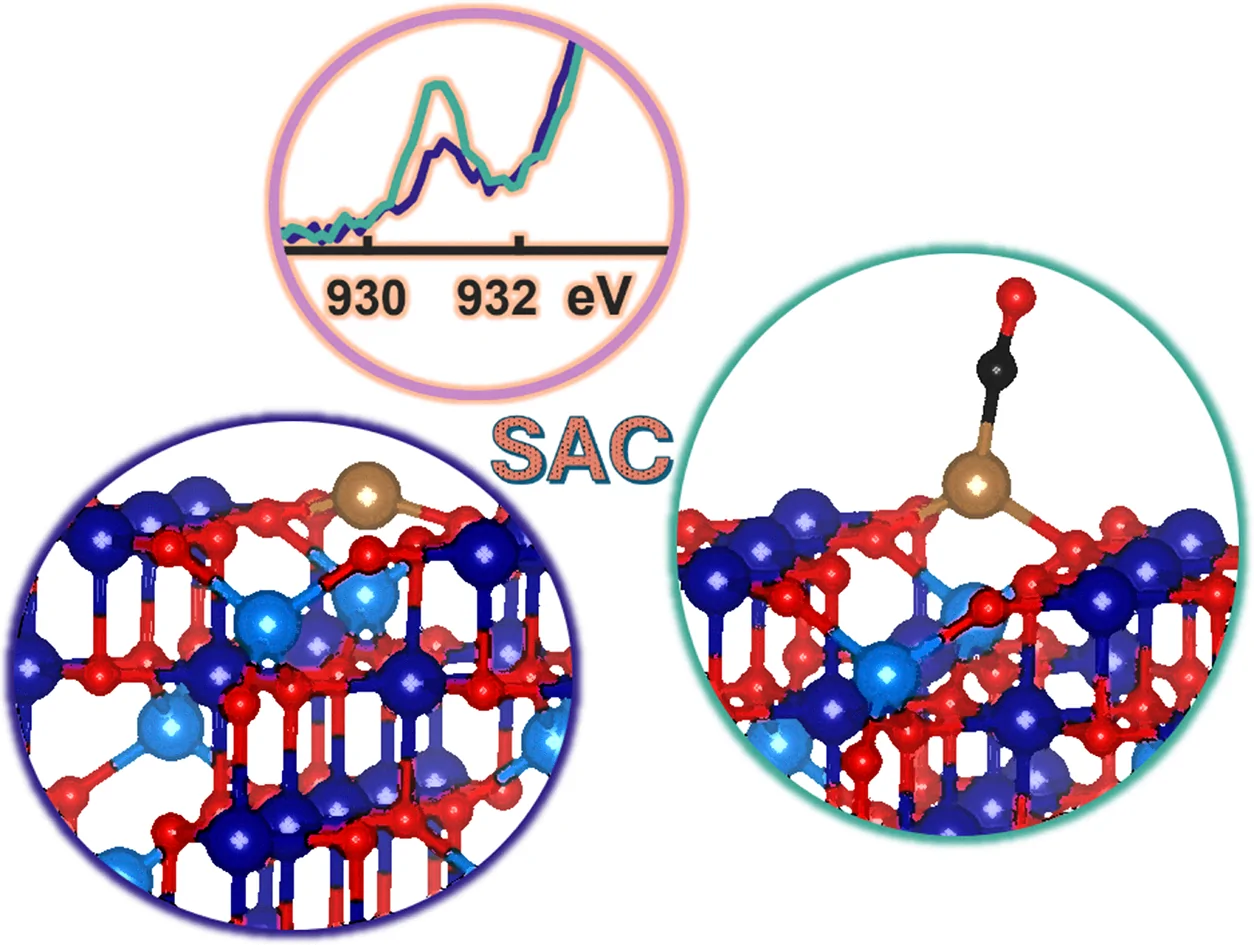

Over the past decade, single atom catalysts (SACs) have become a popular research topic. Where traditional heterogeneous catalysts suffer from poor utilisation of (typically) precious metal, downsizing the active element to individual metal atoms maximises efficiency.^1–5^ However, the properties of SACs differ significantly from those of the parent metal. For example, the electronic structure of the metal atom is modified through the formation of chemical bonds with the support lattice and the local geometry of the active site is known to play a significant role, prompting comparisons with the mononuclear complexes used in homogeneous catalysis.^6,7^ The bonds to the support are then considered “surface ligands”. Provided the active atoms occupy the same site on the support, one can expect the advantages of homogeneous catalysis such as well-defined structure, high activity and high selectivity in addition to ease of separation from the catalytic product.^2,3,5^ Indeed, this has been reported for hydroformylation of alkenes, which is the most widespread industrial application of homogeneous catalysis.^8,9^

Unfortunately, it remains difficult to ascertain, let alone control, the atomic-scale structure of SACs.^10^ Many studies utilise X-ray absorption near-edge spectroscopy (XANES) measurements, but interpretation of these data is not trivial.^11–13^ Spectra obtained from the SAC are typically compared to a bulk standard, which is problematic because the local structure of the metal atoms must be different. To be active in catalysis, the SAC must reside at the support surface and should therefore be undercoordinated compared to an atom residing in the bulk of a metal oxide sample. However, such an undercoordinated, reactive metal atom will inevitably adsorb molecules from the environment, thereby altering the XANES spectrum.

To investigate how the aforementioned factors affect XANES spectra we applied this technique to the study of a well-defined, model SAC: Cu_1_/Fe_3_O_4_(001).^14^ The system takes advantage of a special the 
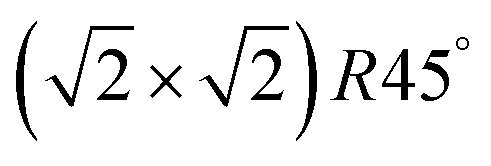
 surface reconstruction (Fig. 1) that stabilizes metal adatoms.^15,16^ These adatoms are comparatively stable, with some species observed to be resistant to agglomeration up to temperatures as high as 700 K.^17,18^

**Fig. 1:**
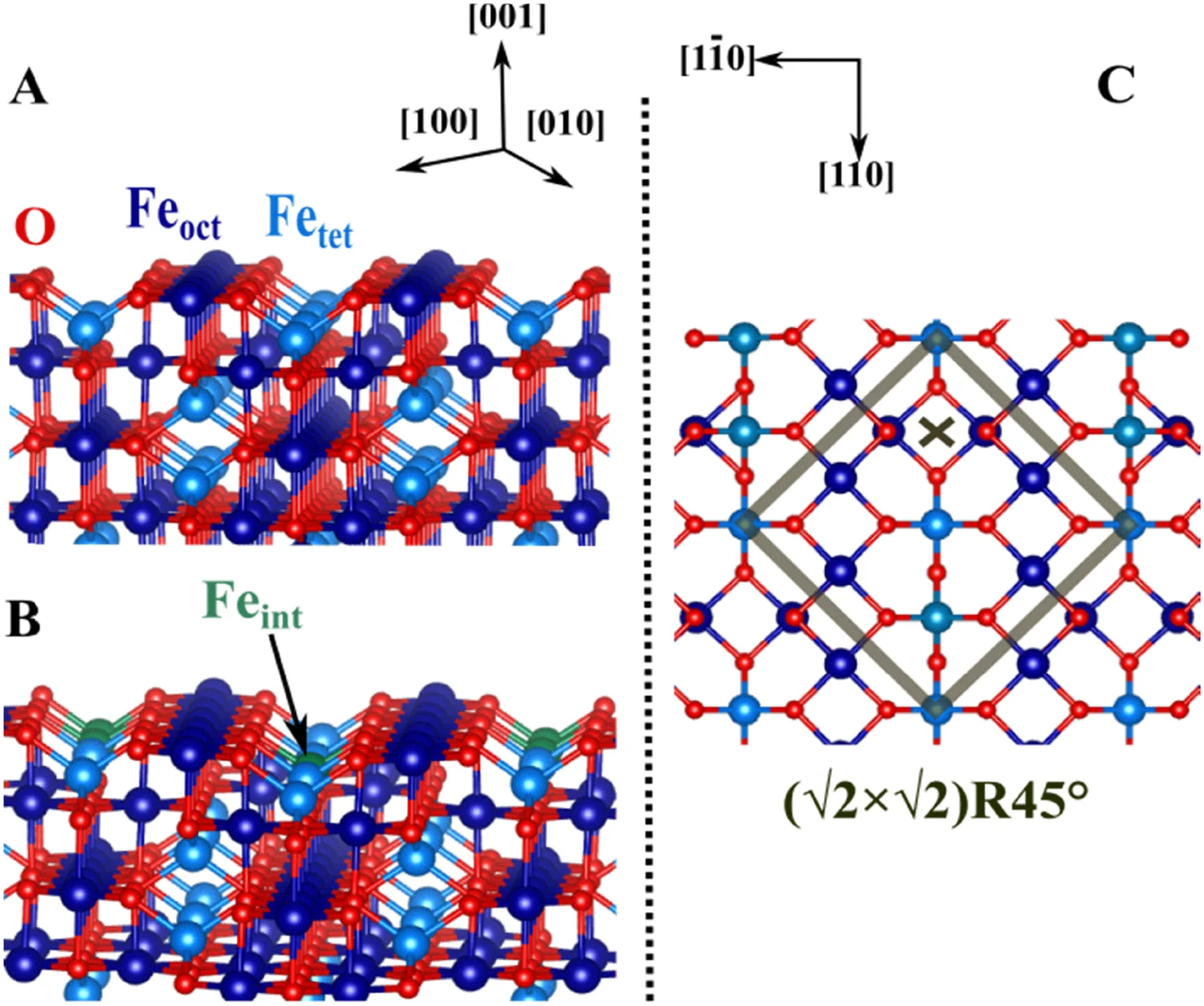
(A) Perspective view of the bulk structure of Fe_3_O_4_, with oxygen, O, shown in red, octahedral iron, Fe_oct_ in dark blue and tetrahedral iron, Fe_tet_ in light blue. (B) Perspective view of the subsurface cation vacancy structure, with the interstitial Fe_tet_ atoms from the second layer—labelled Fe_int_—replacing the Fe_oct_ pairs from the third layer. (C) Plan view of the reconstructed 
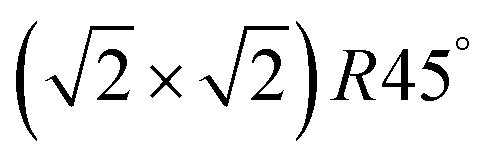
surface, with the cross indicating a missing second-layer Fe_tet_ atom.

The adsorption of various atoms on the Fe_3_O_4_(001) adatom template was previously studied using a combination of techniques including temperature programmed desorption (TPD), X-ray photoelectron spectroscopy, scanning tunnelling microscopy (STM) and normal incidence X-ray standing waves (NIXSW).^14,17–25^ STM measurements suggested most metal adatoms initially occupy bulk-continuation sites, two-fold coordinated to surface O atoms (shown as a cross in Fig. 1C).^14,18–22^ Owing to the uniformity of the system, an unambiguous determination of the Ag, Cu and Ni adatom heights above the surface was achieved by NIXSW^14,24^ which has ultimately confirmed the predictions of DFT calculations.^21^ Following the assertion of the on-surface structure, it has become possible to study trends in the adsorption of molecules, and gain insights into the structure–function relationships in SAC.^10^

Given its importance as a reactant and a poison in many catalytic processes, CO has long been utilised as a probe molecule to study the properties of heterogeneous catalysts.^26–28^ Recent studies of CO adsorption on Fe_3_O_4_(001)-supported SACs^17,20–23,25^ show that CO produces both electronic and structural changes, which in turn affect the adsorption energy. In the extreme case of Pd^23^ and Pt,^17,20^ CO adsorption weakens the metal–O bond and destabilizes the adatom leading to sintering at room temperature.^21^ While this does not occur for the group 11 metals, prior NIXSW studies show that CO adsorption results in Ag adatoms being pulled from the magnetite surface by roughly 0.2 Å, relative to the bare Ag atoms.^25^ A similar *trans* effect^29^ can be expected for Cu adatoms on Fe_3_O_4_(001), which we study here using XANES.

Cu L-edge XANES were obtained from the FlexPES beamline^30^ at MAX IV (Sweden) in the partial electron yield mode for improved sensitivity to the dilute amounts of Cu. Cu was deposited onto the as-prepared Fe_3_O_4_(001) sample in ultra-high vacuum conditions using an e-beam evaporator, with the evaporation rate monitored by a water-cooled quartz crystal microbalance. The surface cleanliness was ascertained by soft X-ray photoelectron spectroscopy and preparation of the 
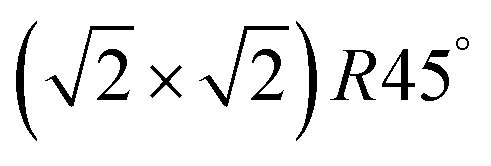
 reconstruction was established by low energy electron diffraction. Further experimental and computational details are available in the (SI).

The Cu L-edge XANES of Cu/Fe_3_O_4_(001) is shown in Fig. 2A, compared against reference spectra from a clean Cu(111) single crystal and a Cu_2_O(100) single crystal. A reference spectrum was measured from the Cu_2_O(100) crystal both as loaded from air (Cu_2_O), and after a 10 minute (Ar^+^) sputtering cycle (Cu_2_O-s). The spectrum acquired from the Cu/Fe_3_O_4_(001) sample resembles that from the as-loaded Cu_2_O spectrum. This result is expected because in bulk Cu_2_O, Cu^1+^ cations are two-fold coordinated to O^2−^ anions (see Fig. 3A) which is similar to the determined structure of the Cu adatoms on the Fe_3_O_4_(001) surface. Both spectra exhibit a pre-edge feature at 931 eV. Interestingly, the pre-edge feature disappears from the Cu_2_O reference upon sputtering and is significantly attenuated in the less surface-sensitive fluorescence and total electron yield measurements (see Fig. S2 in the SI).

**Fig. 2:**
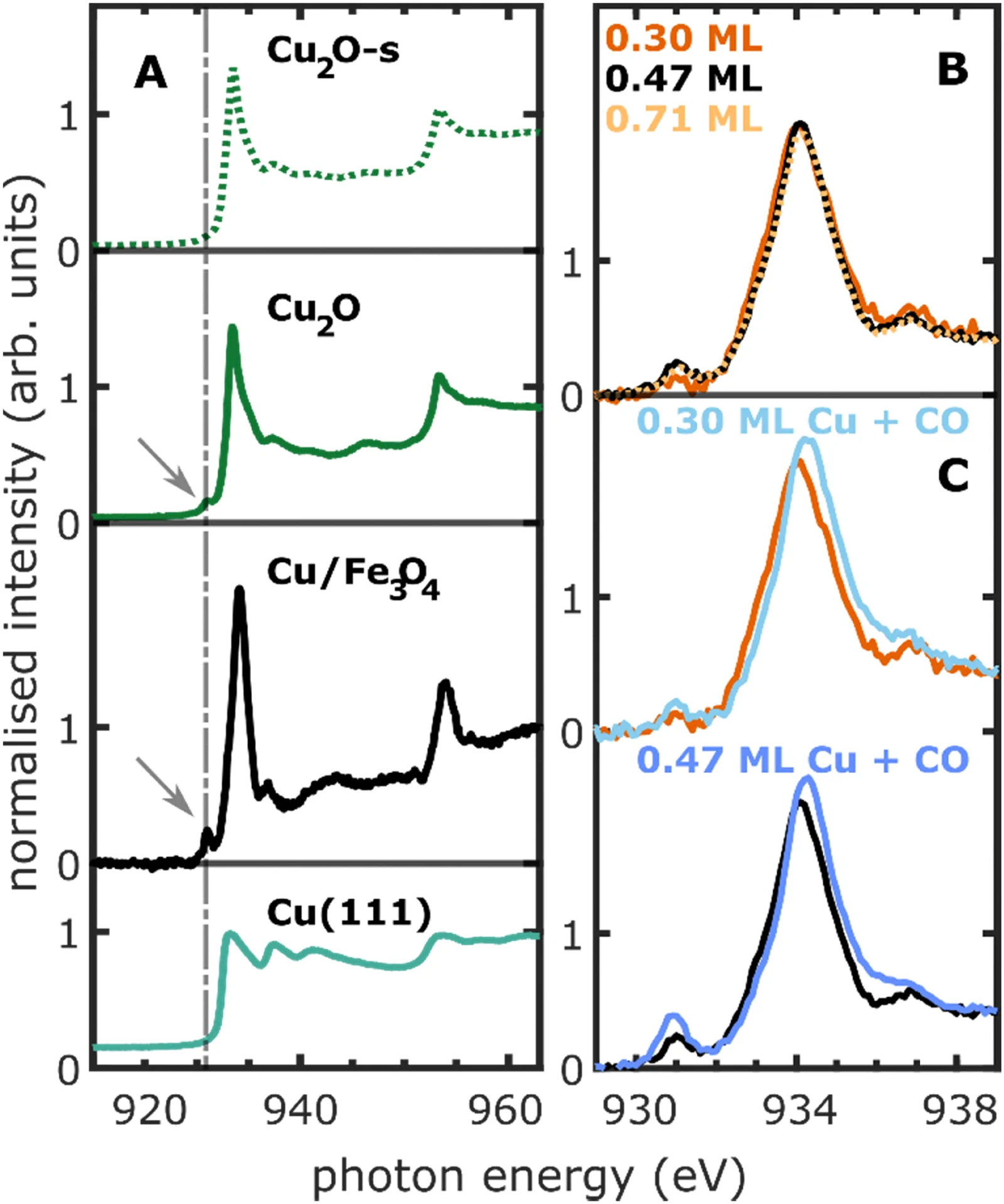
(A) Comparison of the Cu L-edge XANES acquired from 0.47 ML of Cu deposited on Fe_3_O_4_(001), bulk Cu(111) and bulk Cu_2_O(100). The Cu_2_O(100) XANES were measured before (Cu_2_O) and after (Cu_2_O-s) sputtering. The arrows indicate a pre-edge feature located at roughly 931 eV (vertical cursor). (B) The evolution of the Cu L-edge XANES as the Cu coverage on the Fe_3_O_4_(001) surface is increased from 0.30 ML up to 0.71 ML. (C) The effect of 6.75 L CO exposure on the Cu L-edge XANES on 0.30 ML (top) and 0.47 ML (bottom) of Cu adsorbed on Fe_3_O_4_ (001).

**Fig. 3:**
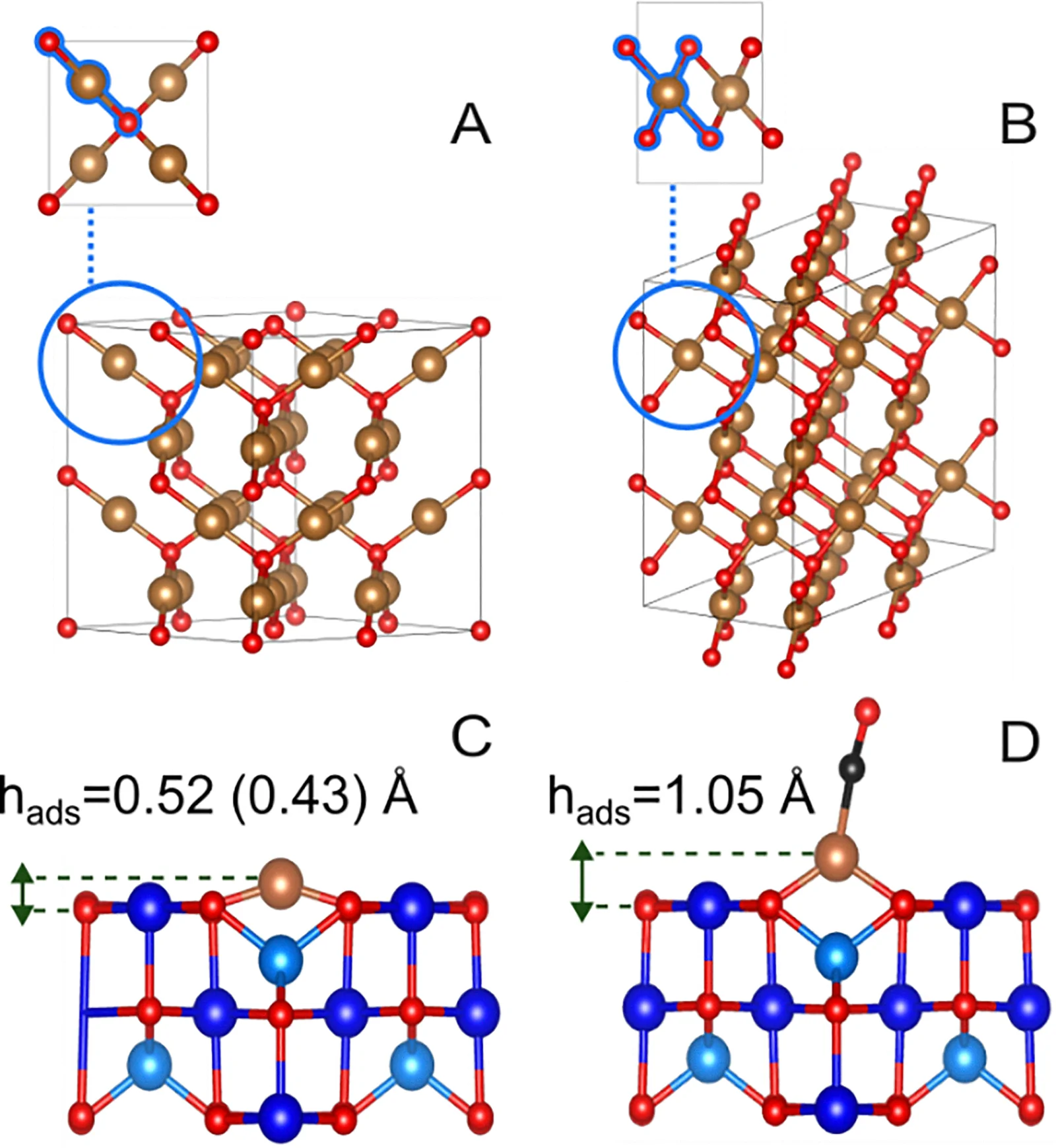
The 2 × 2 × 2 supercell of (A) Cu_2_O and (B) CuO. The blue circles highlight the (A) Cu(i) two-fold and (B) the Cu(ii) four-fold coordination to lattice O atoms. The inset along the [100] direction highlights the Cu atom coordination (blue outlines) in the metal oxide unit cell. (C) The Cu adsorption site on the Fe_3_O_4_ (001) surface with a nearly linear coordination geometry and (D) the three-fold coordination geometry upon ligation to CO. The adsorption heights, *h*_ads_, are quoted from previously published DFT calculations,^21^ whereas the NIXSW measurement yields the average Cu adatom height as 0.43 Å.^14^

In the Cu_2_O data, the suppression of the pre-edge feature suppressed in the bulk, and its removal by sputtering, suggests it is likely related to surface contamination. Indeed, the pre-edge feature at 931 eV in Cu L-edge XANES of Cu_2_O has been previously reported in the literature and ascribed to contamination by common Cu(ii) impurities such as carbonates or hydroxides.^31^ In the Cu/Fe_3_O_4_(001) measurements, varying the Cu adatom coverage does not produce a significant effect on the absorption spectra (see Fig. 2B), with only modest differences observed at low coverage—attributed to a poorer signal-to-noise ratio. The coordination of CO to the Cu adatom results in only minor changes to the spectrum above 931 eV (Fig. 2C), but a significant increase in the intensity of the pre-edge feature at 931 eV. This suggests the pre-edge feature arises due to the minute differences in coordination between Cu atoms in Cu/Fe_3_O_4_(001) and Cu_2_O, which are accentuated by CO coordination. For the two different Cu adatom coverages studied (Fig. 2C), the adsorption of CO positively shifts the Cu L_3_ edge of Cu/Fe_3_O_4_(001) XANES by 0.2 eV. Such a shift in the d-states implies a strongly bound copper mono-carbonyl, as indicated by previously published TPD.^21^ Therefore, the remainder of the discussion will focus on identifying potential mechanisms for the formation of this feature at 931 eV as well as potential implications that can be drawn from its alteration by CO ligation.

The linear O–Cu–O units of the Cu/Fe_3_O_4_(001) surface can be approximated as a 3d^10^ oxide with the valence band comprising the filled Cu 3d states sitting above the O 2p states and the conduction band comprising the unfilled Cu 4s states sitting below the empty Cu 4p states. Based on symmetry, the O 2p orbitals can hybridise with a Cu 3d_*z*^2^_ orbital *via* a σ-overlap, and with the Cu 3d_*xz*_ and 3a_*yz*_ orbitals *via* a π-overlap. Therefore, the valence band of the oxide-supported Cu adatom will likely comprise the filled Cu 3d_*xz*_, d_*yz*_ and d_*z*^2^_ states mixed to some extent with the O 2p states, leaving the Cu 3d_*xy*_ and d_*x*^2^−*y*^2^_ orbitals non-bonding and localised on Cu. Several DFT based studies^32–34^ also point to an intra-atomic Cu 3d–4s hybridisation consistent with a non-negligible 3d_*z*^2^_ contribution in the Cu_2_O conduction band and a 4s character in the valence band.

Often, CO ligands coordinate to transition metals through a dominant mechanism of 3dπ → 2pπ* back-bonding for which to occur the CO molecule should first form a σ bond with the metal. Thus, while the 3d_*z*^2^_ contribution to the 4s and 4p charge is reported to be marginal,^32^ the formation of a σ bond may be further encouraged *via* a lone-pair donation from an occupied CO 2pσ orbital to either an unfilled Cu 4sσ orbital or even an unoccupied Cu 3d_*z*^2^_σ* orbital that has lost charge to the conduction band. Regardless of the exact σ-donation mechanism, CO ligation along the *z*-axis will most likely result in strong π interactions between the CO 2pπ* and the Cu 3d_*xz*_ and d_*yz*_ orbitals as well as weaker σ interactions with the Cu 3d_*x*^2^−*y*^2^_ orbitals. This interpretation is supported by the pre- and post-ligation density of states (DOS) plots (Fig. S3) as described in §3 of the SI. While DOS calculations do not model the core-hole formation of the XANES process and thus, cannot be used to reproduce the observed XANE spectra, they can still be implemented to infer the energetic overlap between the CO and Cu orbitals.

While the pre-edge feature at 931 eV is resolved within the energy range of the Cu L_3_ absorption edge of CuO,^35^ unlike the four-fold coordinated Cu atoms in CuO (Fig. 3B), the Fe_3_O_4_(001) supported Cu adatoms are expected to have a three-fold coordination: a two-fold coordination to the surface O and a one-fold coordination to the CO. It has been shown through STM measurements that the Cu adatom maintains the same adsorption site following CO exposure.^21^ Therefore, much like the CuO impurity in the Cu_2_O crystal leading to a pre-edge structure in Cu L-edge XANES of Cu_2_O, the feature at 931 eV can be linked to further oxidation of the Cu(i) species. Specifically, if the Cu(i) adatoms experience a partial depopulation of the d-states into a 3d^10−*x*^ configuration, with *x* ≪ 1, then the 2p → 3d transitions will be very similar in energy to that of the open-shell Cu(ii), with d^9^ configuration. Therefore, Fig. 2C can be said to indirectly capture the effect of CO adsorption on the Cu/Fe_3_O_4_(001) surface. While the near-linear coordination environment of bare Cu on Fe_3_O_4_(001) resembles that of Cu(i) in Cu_2_O (see Fig. 3A and C), displacement from the linear configuration, towards a bent geometry, may result in a 3d^10−*x*^ electronic configuration. Upon adsorption of CO, the Cu is even further displaced from a linear configuration into a more trigonal geometry, resembling that of Cu(ii) in CuO (see Fig. 3B and D). This distortion may therefore result in a greater depopulation of the d-states, and thus a more intense feature at 931 eV. We have previously shown through XSW measurements of Ag adatoms on Fe_3_O_4_(001), that coordination to CO pulls Ag adatoms from the surface by nearly 0.2 Å.^25^ A similar distortion in the Cu adsorption structure has been predicted by DFT,^21^ driving the linear Cu_2_O-like arrangement towards a more trigonal geometry (Fig. 3C and D). Accurate DFT simulations of L_2,3_-edge XANE spectra are challenging,^36^ as most methods ignore spin–orbit coupling (SOC) or treat SOC by inclusion of an empirical shift. Accurate and reliable SOC-inclusive DFT simulations of XANES for molecular systems is an active field of research,^37^ but is currently out of reach for solids and surfaces. The work presented here provides clear model data for future developments of SOC-inclusive DFT simulations in this field.

The coordination geometry of Cu adatoms on the Fe_3_O_4_(001) surface before and after CO ligation has been studied using XANES. It is found that Cu adatoms produce XANES data very similar to that of Cu_2_O, confirming two-fold coordination to surface O atoms determined in prior work.^14,21^ More notably, a pre-edge feature at 931 eV is present in the Cu/Fe_3_O_4_(001), CO/Cu/Fe_3_O_4_(001) and the Cu_2_O XANES data. In the case of Cu_2_O single crystal, this feature has been attributed to Cu(ii) impurities,^31^ supported by the lack of a pre-edge feature in the Cu_2_O signal post sputtering. For the Cu/Fe_3_O_4_(001) and CO/Cu/Fe_3_O_4_(001) surfaces, we postulate the 931 eV- feature is due to a partial depopulation of the Cu 3d state due to displacement of the O–Cu–O bond away from a linear configuration. Upon adsorption to the Fe_3_O_4_(001) support, the Cu d-state partially depopulates into a 3d^10−*x*^ configuration, with *x* ≪ 1, which results in 2p → 3d transitions presenting at a similar energy range to that of 3d^9^ configuration of Cu(ii) in CuO. In line with this assumption, we observe that, while CO ligation results in minor changes in the XANES data above 932 eV, it strongly enhances the pre-edge feature at 931 eV. This enhancement is assigned to a further depopulation of the d-state by the increased displacement of the Cu atom away from its linear configuration with the (surface) O atoms. In summary, Cu adatoms on the Fe_3_O_4_(001) surface, while mostly sharing the coordination characteristics of Cu_2_O, have a slight CuO character, an effect that is accentuated by carbonyl ligation.

## Conflicts of interest

There are no conflicts to declare.

## Supplementary Material

CC-062-D5CC04046A-s001

## Data Availability

The data supporting this article are available from the University of Nottingham Repository at the following DOI: https://doi.org/10.17639/nott.39748. Supplementary information (SI) includes further experimental and computational details, X-ray photoelectron spectroscopy from the prepared samples, further XANES measurements from our reference sample and the calculated projected density of states. See DOI: https://doi.org/10.1039/d5cc04046a.
