# Supplementary material for: A XANES study of Cu adatom coordination on Fe3O4(001)
Source: Chem Commun (Camb). 2026 Sep 3;62(77):19250–3. doi: 10.1039/d5cc04046a (PMC13548307; doi:10.1039/d5cc04046a)
Supplement: Supplementary file 1 [file CC-062-D5CC04046A-s001.pdf]

Supplementary Information (SI) for Chemical Communications.
This journal is © The Royal Society of Chemistry 2026

A XANES study of Cu adatom coordination on $Fe_3O_4(001)$

Fulden Eratam [a], Lena Haager [§ b], Benedikt P. Klein [§ a c †], Matthew A. Stoodley [§ a c], Matthias Meier[b], Alexei Preobrajenski [d], Stephan Appelfeller [d], Gareth S. Parkinson [b] and David A. Duncan * [a e]

[a] Diamond Light Source, Harwell Science and Innovation Campus, Didcot, OX11 0DE, United Kingdom.

[b] Institute of Applied Physics, TU Wien, Karlsplatz 13, 1040 Vienna

[c] Department of Chemistry, University of Warwick, Gibbet Hill Road, Coventry, CV4 7AL, United Kingdom.

[d] MAX IV Laboratory, University of Lund, Fotongatan 2, 224 84 Lund, Sweden.

[e] School of Chemistry, University of Nottingham, University Park, Nottingham NG7 2RD, United Kingdom

[*] Corresponding author: david.duncan@nottingham.ac.uk

[§] These authors contributed equally.

[†] Current address: Research Center for Materials Analysis, Korea Basic Science Institute, 169-148 Gwahak-ro, Yuseong-gu, Daejeon, 34133, Republic of Korea.

Supporting Information

Experimental Details

The experimental measurements were performed using the ES01 end station at the Surface and Materials Science (SMS) branch of the FlexPES (Flexible Photo Electron Spectroscopy) beamline at MAX IV Laboratory, Sweden.[1] The measured $Fe_3O_4(001)$, Cu(111) and Cu_2O samples were acquired from Surface Preparation Laboratory (SPL), Netherlands. The clean $Fe_3O_4(001)$ surface was prepared using repeated Ar^+ sputtering and annealing cycles (920 K), where alternate annealing cycles were performed either in an oxygen atmosphere (1×10^{-06} mbar) or in vacuum (1×10^{-09} mbar) to avoid undesired oxidation and/or surface reduction. The formation of the desired $(\sqrt{2} \times \sqrt{2})R45°$ reconstruction was confirmed by low energy electron diffraction (not shown), while the cleanliness of the surface was ascertained by soft X-ray photoelectron spectroscopy (SXPS).

Cu adatoms were deposited using an e-beam (Focus EFM 3T) evaporator, whose evaporation rate was monitored by a water-cooled quartz crystal microbalance (QCM) that directly faced the evaporator at roughly the position of the sample. The coverages were defined in monolayers where 1 ML corresponds to one metal atom per $(\sqrt{2} \times \sqrt{2})R45°$ unit cell or 1.42 atoms per nm^2. SXPS was employed to identify the correct coverage of the Cu adatoms deposited (see Figure S1). The sample was exposed to CO by backfilling the chamber through a high precision leak valve while the sample was held at ~ 150 K, above the CO desorption temperature on $Fe_3O_4(001)$.[2] The Cu / $Fe_3O_4(001)$ surface was exposed to 6.75 L of CO, with 1 L corresponding to 10^{-06} mbar·s. In preparation for the reference spectra, the Cu(111) surface was cleaned in ultrahigh vacuum using conventional Ar^+ sputtering and annealing cycles, whereas the Cu_2O surface was cleaned by Ar^+ sputtering only.

The SXPS data were acquired using a DA30-L (Scienta Omicron) hemispherical analyser. For optimal surface sensitivity, the NEXAFS measurements were performed in the partial electron yield (PEY) detection mode using a microchannel plate (MCP) with an installed retardation grid, placed underneath the sample at a small angle from the horizontal plane. A retardation voltage of 670 V was used to retard a high percentage of the inelastically scattered electrons. Partial fluorescent yield (PFY) and total electron yield (TEY) modes were also acquired. The PFY data was acquired using a silicon-drift detector located in the plane orthogonal to the beam direction, whereas the TEY data was acquired via the drain current from the sample. For the NEXAFS measurement, the sample was orientated such that the angle between the photon polarisation and the surface normal was ~53°, roughly corresponding to the magic angle.

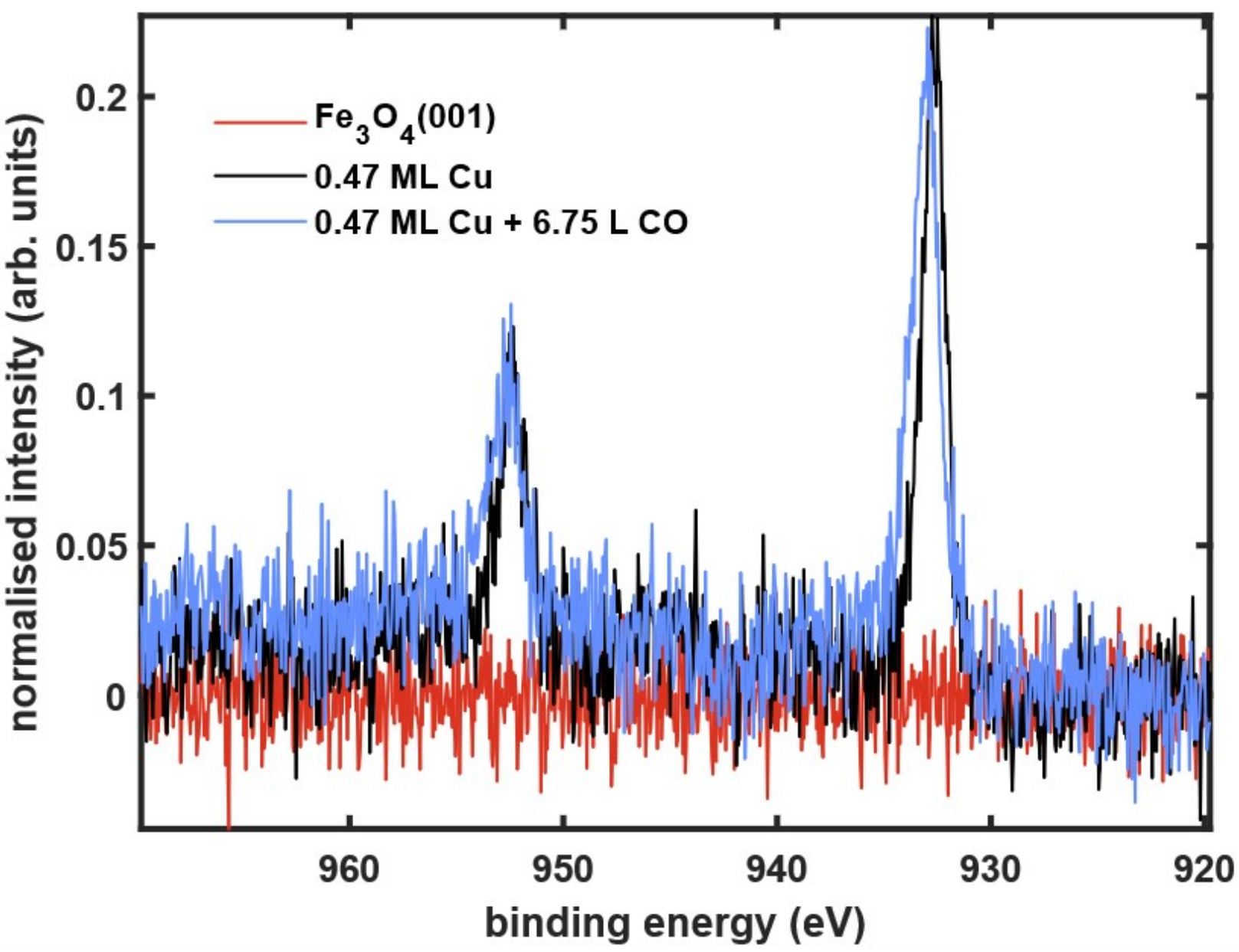

Figure S1 The Cu 2p XP spectrum obtained from the clean $Fe_3O_4(001)$ surface, compared to XP spectra produced with 0.47 ML Cu coverage, before (black) and after the adsorption of CO (blue). Following CO ligation, both the Cu $2p_{1/2}$ peak (950-955 eV region) and the Cu $2p_{3/2}$ peak (930-935 eV region) experiences a positive shift (~ 0.2-0.4 eV). In both cases, the positive shift can be attributed to the strong π^* back-bonding between the Cu adatoms and the ligated CO molecules.

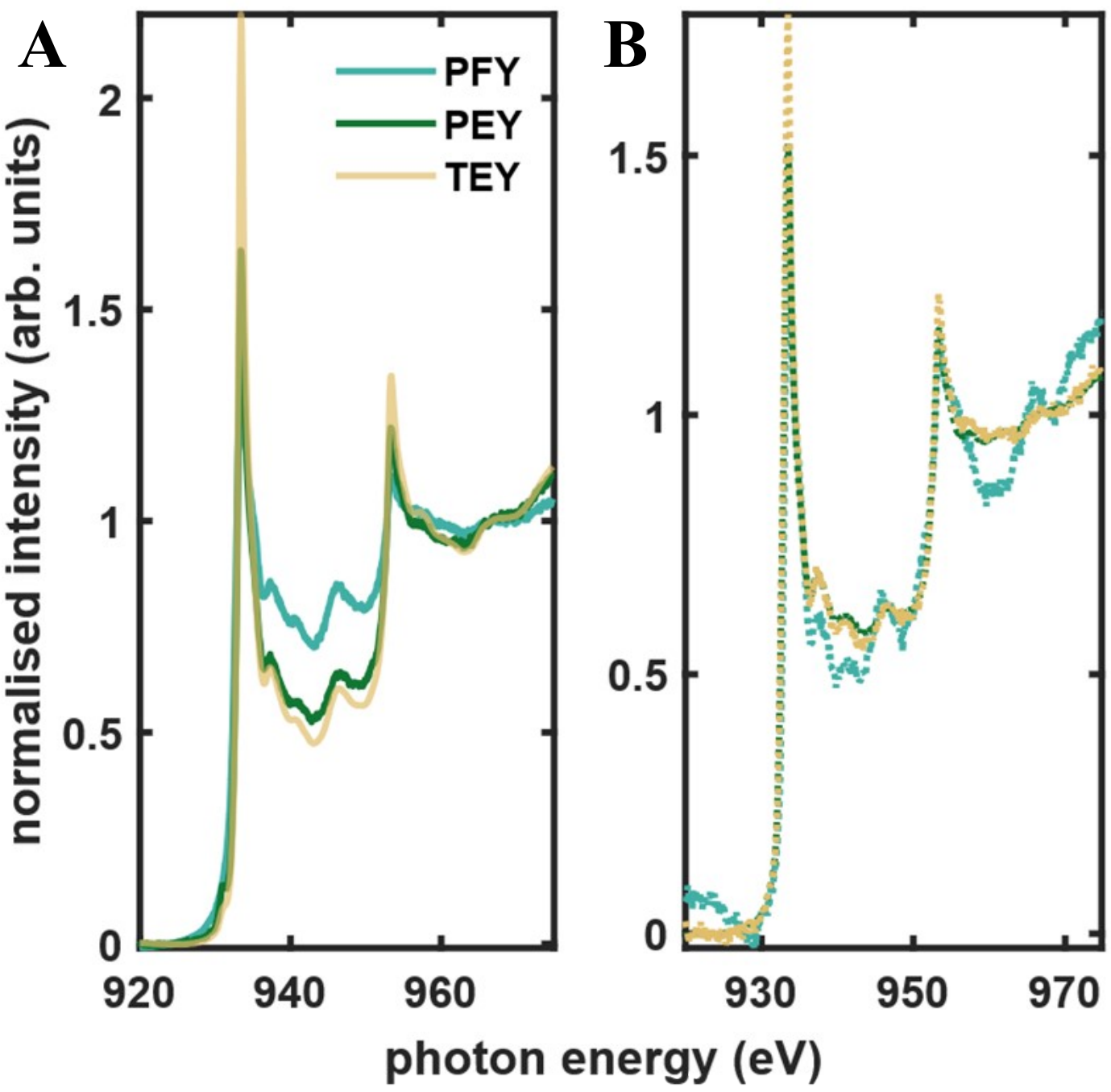

Figure S2 Comparisons of partial fluorescent (PFY), partial electron (PEY) and total electron yield (TEY) spectra obtained for the Cu_2O reference before (A) and after cleaning (B). The pre-edge feature at 931 eV is only resolved in the case of PEY spectra taken from the air loaded Cu_2O sample. The disappearance of the 931 eV-peak in the clean spectra is consistent with this feature resulting from a Cu(II) impurity present in the Cu_2O single crystal.

The Cu/ Fe_3O_4(001) projected density of states (PDOS) pre- and post- CO ligation

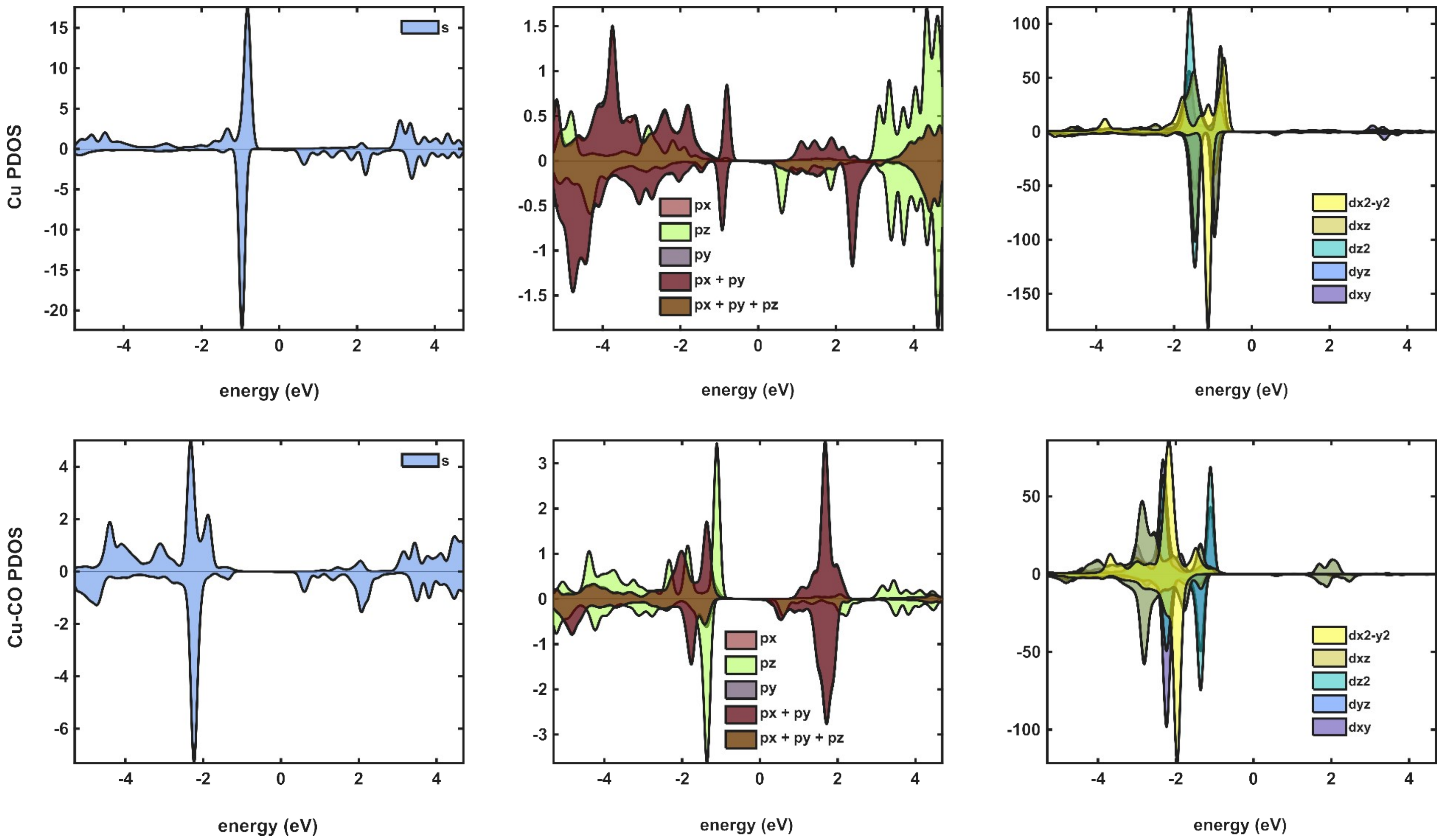

Figure S3: Depicted are the projected density of states (PDOS) calculated before (top row) and after (bottom row) CO ligation to Cu adatoms on the Fe_3O_4(001) surface. Note the significant overlaps between the p_y and p_x orbitals make the p_y density of states very difficult to discern.

Focusing on the d-states first, the π* back-donation from the Cu 3d orbitals results in an energy shift of the d-states towards the CO states consistent with the Cu 3d – CO 2pπ* orbital overlap. Consequently, the broader energy distribution across the Cu 3d states is now focussed towards the 2pπ* state of the CO molecule, both in the conduction and the valance bands. The overall picture is consistent with the assumption that there are weaker σ interactions with the Cu $3d_{z^2}$ and $d_{x^2-y^2}$ orbitals in the valance band compared to the stronger π interactions with the Cu 3d_{xz} and d_{yz} near or within the conduction band.

While the $3p_y$ and $3p_x$ orbitals undergo a shift which concentrates the distribution near ~ 2 eV in the conduction band, the $3p_z$ orbital experiences an increase in density near -2 eV in the valance band.

The Cu 4s states also undergo a significant energy shift which is more pronounced in the valance band. Indeed, the 4s states appear to be shadowing the Cu 3d states as may be expected from the local Cu 3d-4s hybridisation. What is striking, however, is the depletion in the 4s density of states (even when accounting for the broadening) which suggests a more dynamic involvement in the π* back-donation of charge.

References

1. A. Preobrajenski, A. Generalov, G. Öhrwall, M. Tchaplyguine, H. Tarawneh, S. Appelfeller, E. Frampton and N. Walsh, *Synchrotron Radiation*, 2023, **30**, 831-840.
2. J. Hulva, M. Meier, R. Bliem, Z. Jakub, F. Kraushofer, M. Schmid, U. Diebold, C. Franchini and G. S. Parkinson, *Science*, 2021, **371**, 375-379.
